# miR-339-3p promotes AT1-AA-induced vascular inflammation by upregulating NFATc3 protein expression in vascular smooth muscle cells: miR-339-3p aggravates vascular inflammation

**DOI:** 10.3724/abbs.2023009

**Published:** 2023-02-24

**Authors:** Yang Li, Yaolin Long, Xiaoyan Zhi, Haihu Hao, Xiaohui Wang, Huirong Liu, Li Wang

**Affiliations:** 1 School of Basic Medical Sciences Shanxi Medical University Taiyuan 030001 China; 2 Department of Orthopedics Shanxi Bethune Hospital & Shanxi Academy of Medical Sciences Taiyuan 030032 China; 3 School of Basic Medical Sciences Capital Medical University Beijing 100069 China

**Keywords:** angiotensin II-1 receptor autoantibody (AT1-AA), miR-339-3p, NFATc3, vascular inflammation

## Abstract

Vascular inflammation induced by angiotensin II-1 receptor autoantibody (AT1-AA) is involved in the occurrence and development of various cardiovascular diseases. miR-339-3p is closely related to the degree of vasodilation of aortic aneurysm and is also involved in the occurrence and development of acute pancreatitis. However, it is still unclear whether miR-339-3p influences AT1-AA-induced vascular inflammation. In this study, the role and mechanism of miR-339-3p in AT1-AA-induced vascular inflammation are studied. RT-PCR detection shows that the miR-339-3p levels in the thoracic aorta and serum exosomes of AT1-AA-positive rats are significantly increased. The miRwalk database predicts the mRNAs that miR-339-3p can bind to their 5′UTR. Subsequently, it is found that the number of genes contained in the T cell receptor pathway is high through KEGG analysis, and NFATc3 among them can promote the secretion of various inflammatory cytokines. AT1-AA-induced upregulation of miR-339-3p expression in vascular smooth muscle cells (VSMCs) can lead to a significant increase in NFATc3 protein level and promote vascular inflammation. Inhibition of miR-339-3p with antagomir-339-3p can significantly reverse AT1-AA-induced high expressions of IL-6, IL-1β and TNF-α proteins in rat thoracic aorta and VSMCs. That is, AT1-AA can upregulate the expression of miR-339-3p in VSMCs, and the increased miR-339-3p targets the 5′UTR of NFATc3 mRNA to increase the protein level of NFATc3, thereby aggravating the occurrence of vascular inflammation. These findings provide new experimental evidence for the involvement of miRNAs in regulating vascular inflammatory diseases.

## Introduction

In recent years, despite the continuous improvement in the treatment and intervention of cardiovascular diseases, the status quo of the high morbidity and mortality of human cardiovascular diseases in the world has not changed [1]. One of the main causes of cardiovascular disease is chronic inflammatory disease [2]. Inflammation of the blood vessel wall runs through almost the whole process of cardiovascular disease [3].

Pathological changes such as increased blood pressure and inflammation of the vessel wall have been reported after activation of the renin-angiotensin-aldosterone system (RAAS) [4]. Studies have shown that angiotensin II-1 receptor autoantibody (AT1-AA), which is widely present in patients with inflammatory diseases, can significantly and continuously activate the RAAS and then release tumor necrosis factor-α (TNF-α), interleukin-6 (IL-6) and other proinflammatory cytokines [5], exerting downstream inflammatory injury effects. However, the mechanism of AT1-AA-induced vascular inflammation is not fully understood.

MicroRNAs (miRNAs) have emerged as novel molecular regulators of many genes and pathways involved in normal immune responses, inflammation and autoimmune diseases [6]. miRNAs regulate genes in all steps of the renin-angiotensin-aldosterone signaling (RAAS) system, ultimately affecting protein expression [7]. Studies have shown that miR-339-3p is closely associated with aortic dilation under the aneurysm in patients with high-risk cardiovascular disease [8]. In addition, overexpression of miR-339-3p inhibited cell inflammation in caerulein-induced acute pancreatitis by modulating TRAF3 expression via the p38 pathway [9]. However, it is still unclear whether miR-339-3p influences AT1-AA-induced vascular inflammation.

Therefore, this study focused on exploring the possible mechanism by which miR-339-3p regulates AT1-AA-induced vascular inflammation.

## Materials and Methods

### Establishment of model animals

Male Sprague-Dawley rats (approximately 200 g, 8 weeks old) purchased from the Experimental Animal Center of Shanxi Medical University (Taiyuan, China) were used in the experiment. Extracellular second ring of angiotensin II-1 receptor (AT1R-ECII; GL Biochem, Shanghai, China) was injected subcutaneously (0.4 μg/g) into the back neck every two weeks to complete active immunization, which lasted for three months. Six rats were included in each group. The systolic and diastolic blood pressures of the rat tail were detected using a small animal blood pressure monitor (BP-98A; Softron, Beijing, China). Finally, the blood of model rats was obtained after intraperitoneal injection of 20% pentobarbital sodium at a dose of 40 mg/kg. All animal experiments in this study were approved by the Ethics Committee of Shanxi Medical University (AEEI-2022-010) and conformed to the National Experimental Animal Usage Regulations.

### Extraction of serum exosomal RNA

Exosomes were first extracted using an Exosome Extraction & RNA Isolation Kit (EXORNA30A-1; RENGEN BIOSCIENCES, Shenyang, China). The frozen serum samples were dissolved at room temperature, exosomes with higher purity were extracted through standing, centrifugation, precipitation, and resuspension. The particle size range of the extracted exosomes was detected by nanoparticle tracking technology [10]. Finally, the lysate, chromatography column and eluent were used to extract the RNA wrapped in the exosomes. The operation process was carried out in full accordance with the manufacturer’s instructions.

### Isolation and culture of vascular smooth muscle cells

The SD rats were fixed after anaesthesia, and the thoracic aortas were obtained and immediately placed in prechilled DMEM (8122020; Gibco, Carlsbad, USA). Then, vascular tissues without the outer and inner membranes were cut into smaller pieces in a dry petri dish, and the shredded vascular tissue pieces were evenly and tightly attached to the bottom surface of the cell culture flask. Vascular tissue pieces in the culture flask were incubated in the incubator for 3–6 h, and then approximately 5 mL of 10% FBS medium was added to the culture flask, the medium slowly immersed the tissue pieces, and the incubator was left to culture for 3–4 days. The medium was changed, and the cells were subcultured after approximately one week.

### Cell treatment

Vascular smooth muscle cells (VSMCs) were cultured in DMEM low-glucose medium containing 10% FBS and subcultured normally. VSMCs were treated with 1×10 ^–7^ M AT1-AA for 24 h. After that, the cells were collected for the detection of NFATc3 protein expression and miR-339-3p content. In this study, antagomir-339-3p (5′-UGGCUCUGUCGUCGAGGCGCUCA-3′, GenePharma, Suzhou, China) was used to inhibit the level of miR-339-3p in AT1-AA positive rats and AT1-AA treated VSMCs. Each rat was injected with about 20 nmol antagomir-339-3p through the rat tail vein. Antagomir-339-3p was used in cells at a final concentration of 37.5 nM.

### Transmission electron microscopy

The resolution of exosomes under transmission electron microscopy is 0.1–0.2 nm, which is suitable for the observation of the ultrastructure of exosomes bilayer capsules. It can be observed whether there is exosome-like structure in samples (usually saucer-type or hemispherical with one side depression), and the size of exosomes can be measured at the same time. We submitted the cell supernatant to the company (Enzekangtai, Beijing, China) and extracted exosomes from the samples by ultrafast centrifugation. Exosome suspensions of different concentrations were added to carbon coated copper mesh, stained with uranium dioxyacetate, dried, observed and photographed under a transmission electron microscope (H-7650; Hitachi, Tokyo, Japan).

### Nanoparticle tracking technology analysis (NTA)

Particle size and particle concentration of exosome solutions with particle concentrations from 1×10 ^7^ cells/mL to 1×10 ^9^ cells/mL were detected using ZetaView PMX 110 (Particle Metrix, Meerbusch, Germany). A 60-s video was filmed at a frame rate of 30 frames per second under excited light waves with a wavelength of 405 nm. The particle trajectory was analyzed using NTA software (ZetaView 8.02.28) to obtain the size and concentration of exosomes.

### Real-time PCR (RT-PCR)

RT-PCR was performed to measure the content of miR-339-3p in VSMCs treated with AT1-AA or vascular tissue of AT1-AA-positive rats. E.Z.N.A.® miRNA Isolation kit (R6842-01; OMEGA Biotek Inc., Norcross, USA) was used to extract total microRNA. Then, RNA was reverse transcribed into cDNA using a PCR quantitative kit for miRNA (E22001; GenePharma). Using cDNA as template, fluorescence quantitative PCR was performed according to the manufacturer’s instructions. An automated process of repeated cycles (usually 45) of denaturation of the template DNA (at 95°C), annealing of primers to their complementary sequences (at 50°C), and primer extension (at 62°C) are employed for the amplification of target sequence. The content of miR-339-3p in the original sample was calculated and analysed based on 2 ^–ΔΔCt^. The primers used are: miR-339-3p forward 5′-AAATGAGCGCCTCGACGA-3′, reverse 5′-TATGGTTGTTCACGACTCCTTCAC-3′; U6 forward 5′-CGCTTCGGCAGCACATATAC-3′, reverse 5′-TTCACGAATTTGCGTGTCATC-3′. U6 was used as reference gene, the relative expression of miR-339-3p versus U6 was quantified.

### Western blot analysis

Prepared protein samples (10 μg) were subject to 12% SDS-PAGE, followed by electro-transfer onto PVDF membranes. Then the membranes were blocked in 5% skimmed milk for 1 h. Antibodies against exosome markers CD9 (ab92726; Abcam, Cambridge, UK), CD81 (ab109201; Abcam) and calnexin (ab133615; Abcam) were diluted at a ratio of 1:1000. The inflammatory response of vascular tissue and VSMCs was detected using antibodies against NFATc3 (18222-1-AP, 1:1000 dilution; Proteintech, Wuhan, China) and IL-6 (DF6087, 1:500 dilution; Affinity Biosciences, Cincinnati, USA), IL-1β (DF6251, 1:500 dilution; Affinity Biosciences) and TNF-α (ab205587, 1:500 dilution; Abcam). The internal control anti-β-actin primary antibody (TA-09; Zhongshan Jinqiao Biotechnology, Beijing, China) was used at 1:1000 dilution. The PVDF membranes were incubated with the above primary antibodies at 4°C overnight, followed by incubation with HRP-conjugated goat anti-mouse IgG (ZB-2305, 1:4000; Zhongshan Jinqiao Biotechnology) or HRP-conjugated goat anti-rabbit IgG (ZB-2301, 1:4000; Zhongshan Jinqiao Biotechnology) secondary antibodies for 1 h at room temperature. After extensive wash, protein bands were visualized using ultra high sensitivity ECL kit (HY-K1005; MCE, New Jersey, USA), and images were obtained with a chemiluminescence gel imaging system (12003153; Bio-Rad, Hercules, USA).

### Fluorescence *in situ* hybridization

VSMCs were seeded on cell slides with a diameter of 14 mm, treated with AT1-AA for 24 h, washed with PBS and then fixed with 4% paraformaldehyde for 15 min at room temperature. After being soaked with 0.1% Triton X-100 and washed with PBS twice, cells are exposed to 2× SSC about 30 min at 37°C. Then, cell climbing slices were incubated with a mixture of miR-339-3p probes and hybridization buffer (RNA FISH kit; GenePharma) overnight at 37°C in the dark. The next day, after being washed with 2× SSC and 1× SSC separately, cell climbing slices were sealed with the sealing agent containing DAPI in a dark environment, and then fluorescence images were captured as soon as possible.

### Enzyme-linked immunosorbent assay (ELISA)

A 1 mg/mL antigen stock solution of AT1R-ECII antigenic peptide was prepared with coating buffer Na _2_CO _3_ (100 mM). It was diluted to 1 μg/mL with coating buffer and added to a 96-well microtiter plate at 50 μL/well, and the plate was stored in a refrigerator at 4°C overnight. The next day, the plate was leaved at room temperature for 30 min, and then the liquid was completely removed. Then, all wells were blocked with 5% skimmed milk at 37°C for 1 h. The wells were washed three times with PBST. Serum from actively immunized rats was diluted 1:100, added to the wells (100 μL/well). After incubation at 37°C for 1 h, and the plate was washed three times with PBST. HRP-conjugated goat anti-rat IgG (1:3000) secondary antibody was added and incubated at 37°C for 1 h, followed by three times wash with PBST. Chromogenic solution was added and incubated at 37°C for 10 min, 20 min or 30 min, and a microplate reader (SpectraMax 190; Molecular Devices, San Jose, USA) was used to detect the OD values at a wavelength of 405 nm.

### Bioinformatics analysis

The target genes targeted by miR-339-3p were predicted by miRwalk ( http://mirwalk.umm.uni-heidelberg.de/), and then the proteins corresponding to the predicted genes were analyzed by KEGG ( https://www.kegg.jp/) to determine which pathways these target proteins were located in.

### Dual-luciferase reporter assay

HEK-293A cells in logarithmic growth phase were seeded in 24-well plates. When cells were approximately 80% confluent and ready for use, they were cotransfected with the constructed NFATc3-5′UTR reporter gene plasmid and the mimic (sense: 5′-UGAGCGCCUCGACGACAGAGCCA-3′; antisense: 5′-GCUCUGUCGUCGAGGCGCUCAUU-3′) or negative control (5′-UUCUCCGAACGUGUCACGUTT-3′; antisense: 5′-ACGUGACACGUUCGGAGAATT-3′) (200 pmol, GenePharma) of the target miR-339-3p. The cell lysate was collected according to operating procedure of the Dual Luciferase Reporter Assay Kit (DL101-01; Vazyme, Nanjing, China), mixed with the substrate reaction solution in proportion, and the first reading of fluorescence intensity was carried out with a fluorescence microplate reader (SpectraMax iD5; Molecular Devices, San Jose, USA) as soon as possible. After addition of the Renilla substrate working solution to stop the reaction, the second reading of fluorescence intensity was carried out immediately. The ratio of fluorescence intensity of the first and second time is calculated, and the high and low ratios represent whether miR-339-3p plays a accelerative or inhibitory role.

### Statistical analysis

GraphPad Prism software (version 8.0; GraphPad, San Diego, USA) was used for data analysis. Data are presented as the mean± standard error (SE). Comparisons between two groups were analysed using unpaired Student’s *t* test, while comparisons between multiple groups were analysed using one-way ANOVA. All experiments were repeated three times. *P*<0.05 was considered statistically significant.

## Results

### AT1-AA increases the content of miR-339-3p in rat thoracic aorta and in VSMCs

**Figure 1:**
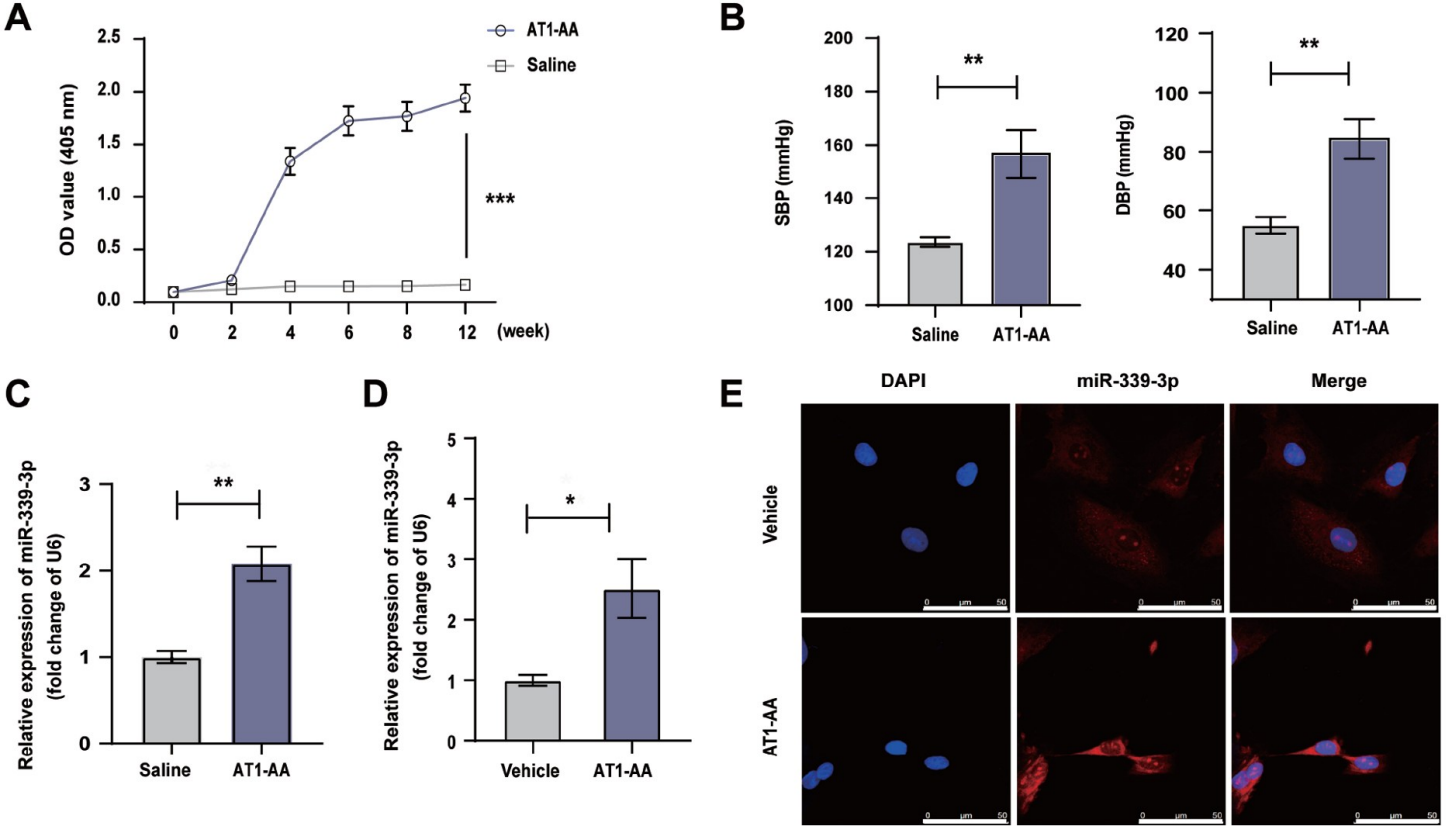
Figure 1 The content of miR-339-3p in the thoracic aortas was detected in AT1-AA-positive rats and AT1-AA-treated VSMCs (A) SD rats were continuously actively immunized with AT1R-ECII for 12 weeks, and the concentration of AT1-AA in rat serum was detected. (B) The systolic and diastolic blood pressures of the rat tail were detected using a small animal blood pressure monitor. (C) Detection of miR-339-3p content in the thoracic aorta of AT1-AA-positive rats and saline group rats by RT-PCR. (D) The content of miR-339-3p in VSMCs treated with AT1-AA for 24 h was detected by RT-qPCR. (E) Fluorescence in situ hybridization was used to detect the changes in miR-339-3p content in VSMCs treated with AT1-AA, Scale bar= 50 μm. Data are presented as the mean±standard error. n=5 or 6. * P<0.05 and ** P<0.01 vs the vehicle group.

After the rats were actively immunized with AT1R-ECII for 12 weeks, the concentration of AT1-AA in the serum of the AT1R-ECII group was significantly higher than that of the saline group ( Figure 1A), indicating that AT1-AA in serum was increased significantly after immunization, and the AT1R-ECII active immunization rat model was successfully established. Furthermore, the systolic and diastolic blood pressures of AT1-AA-positive rats were significantly increased ( Figure 1B). We found that the long-term presence of AT1-AA resulted in a significant increase in miR-339-3p content in the thoracic aorta ( Figure 1C). After treatment of VSMCs with AT1-AA for 24 h, the content of miR-339-3p in the cells was also significantly increased ( Figure 1D), and consistent results were obtained using fluorescence *in situ* hybridization ( Figure 1E). These data indicated that AT1-AA can induce an increase in miR-339-3p expression in the thoracic aorta and in VSMCs.

### The content of miR-339-3p in serum exosomes of AT1-AA-positive rats is significantly increased

**Figure 2:**
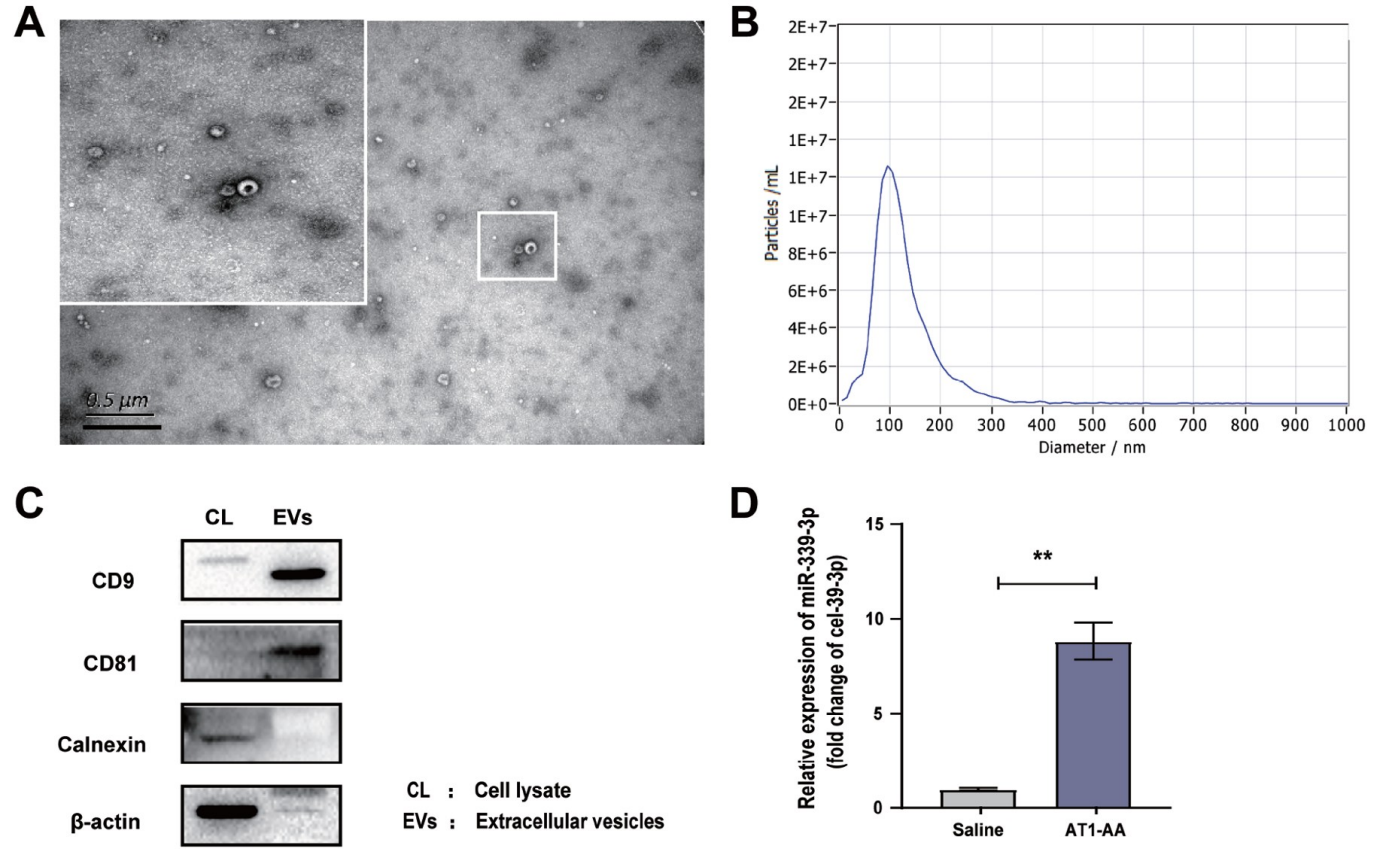
Figure 2 AT1-AA-positive rats were constructed, and the content of miR-339-3p in serum exosomes was detected (A) Rat serum exosomes were extracted, and the morphology of exosomes was observed under a transmission electron microscope, Scale bar= 0.5 μm. (B) The particle size range of the extracted exosomes was detected by nanoparticle tracking technology. (C) Exosome markers were detected by western blot analysis. (D) The serum exosomes of the model rats were extracted, and the content of miR-339-3p was detected by RT-qPCR. Data are presented as the mean±standard error. n=5. ** P<0.01 vs the vehicle group.

After the rats were actively immunized with AT1R-ECII for 12 weeks, the goblet membrane structure of the extracted exosomes was observed under the transmission electron microscope ( Figure 2A), and the diameter of the exosomes was approximately 100 nm ( Figure 2B). It was also found that the canonical exosome proteins CD9 and CD81 were present and the endoplasmic reticulum protein calnexin was absent in the exosomes we isolated and extracted ( Figure 2C). This suggested that we successfully extracted serum exosomes with better purity. The content of miR-339-3p in the serum exosomes of AT1-AA-positive rats was significantly increased compared with that in the control rats ( Figure 2D). These data indicated that, consistent with the increasing trend of miR-339-3p expression in tissue and cells, the content of miR-339-3p in serum exosomes of AT1-AA-positive rats was also increased significantly.

### Inhibition of miR-339-3p can reverse AT1-AA-induced high expression of inflammatory cytokines in rat thoracic aorta and in VSMCs

**Figure 3:**
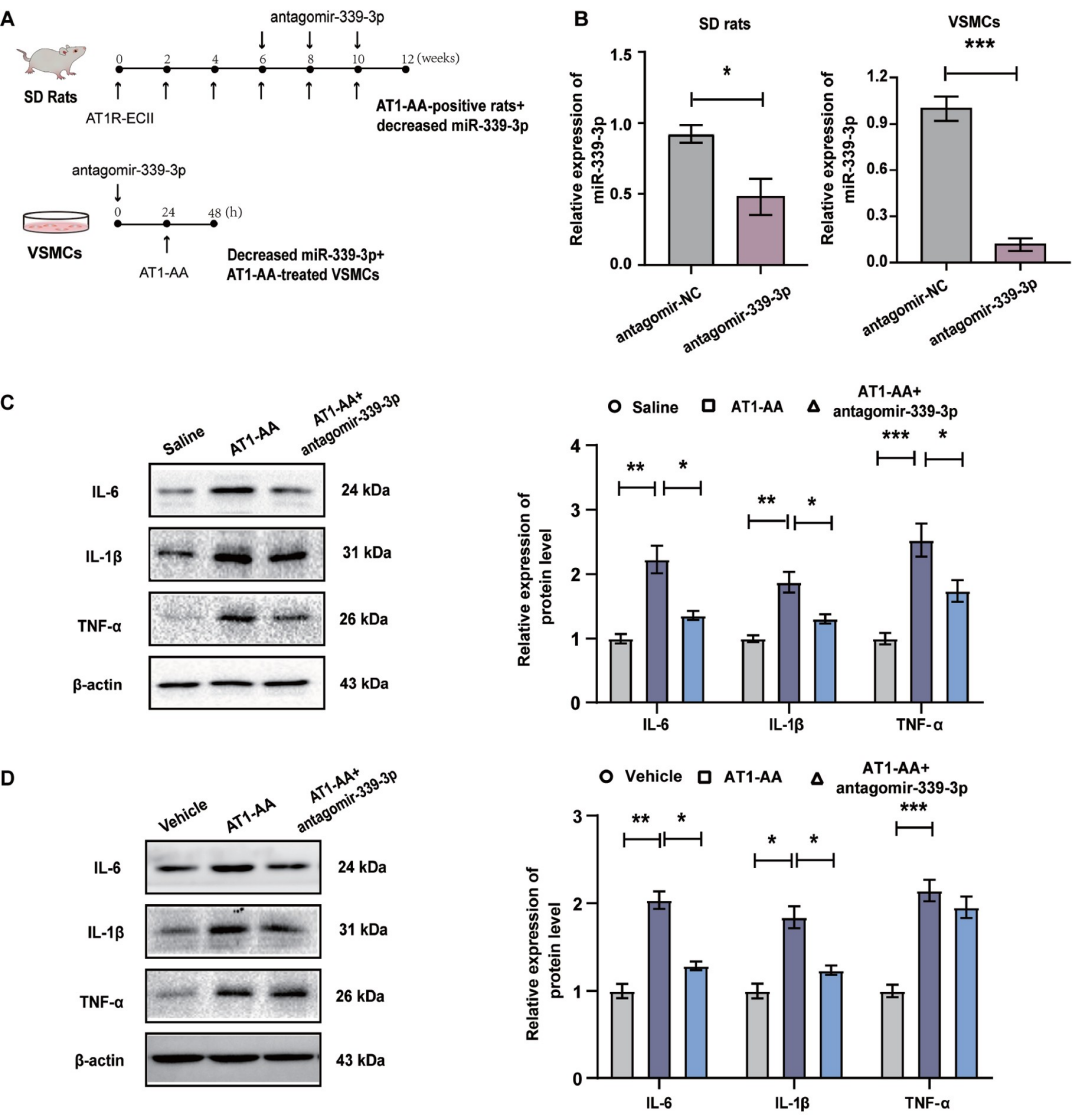
Figure 3 Antagomir-339-3p can reverse AT1-AA-induced high expression of inflammatory cytokines in rat thoracic aorta and VSMCs (A) Experimental design of the inhibition of miR-339-3p by antagomir-339-3p in AT1-AA-positive rats and in VSMCs. (B) Antagomir-339-3p effectively inhibited the content of miR-339-3p in rat thoracic aorta and VSMCs. (C) The protein expression levels of the inflammatory cytokines IL-6, IL-1β and TNF-α in vascular tissue were detected by western blot analysis after inhibiting miR-339-3p in AT1-AA-positive rats. (D) After inhibiting the expression of miR-339-3p, VSMCs were treated with AT1-AA, and the protein expression levels of IL-6, IL-1β and TNF-α in the cells were detected by western blot analysis. Data are presented as the mean±standard error. n=3 or 6. * P<0.05, ** P<0.01 and *** P<0.001.

To further explore the effect of miR-339-3p on the inflammation of vascular tissue and VSMCs, we used antagomir-339-3p to inhibit miR-339-3p *in vivo* and *in vitro* ( Figure 3A,B). The results showed that the protein levels of the inflammatory cytokines IL-6, IL-1β and TNF-α in the thoracic aortas of AT1-AA-positive rats and in VSMCs treated with AT1-AA were significantly increased, compared with the controls. Inhibition of miR-339-3p with antagomir-339-3p significantly reversed the AT1-AA-induced upregulation of IL-6, IL-1β and TNF-α protein expression in vascular tissue ( Figure 3C) and in VSMCs ( Figure 3D). These data indicated that downregulating the content of miR-339-3p reversed the high expression of inflammatory cytokines induced by AT1-AA.

### miR-339-3p efficiently binds to the 5′UTR of *NFATc3* and promotes its protein expression

**Figure 4:**
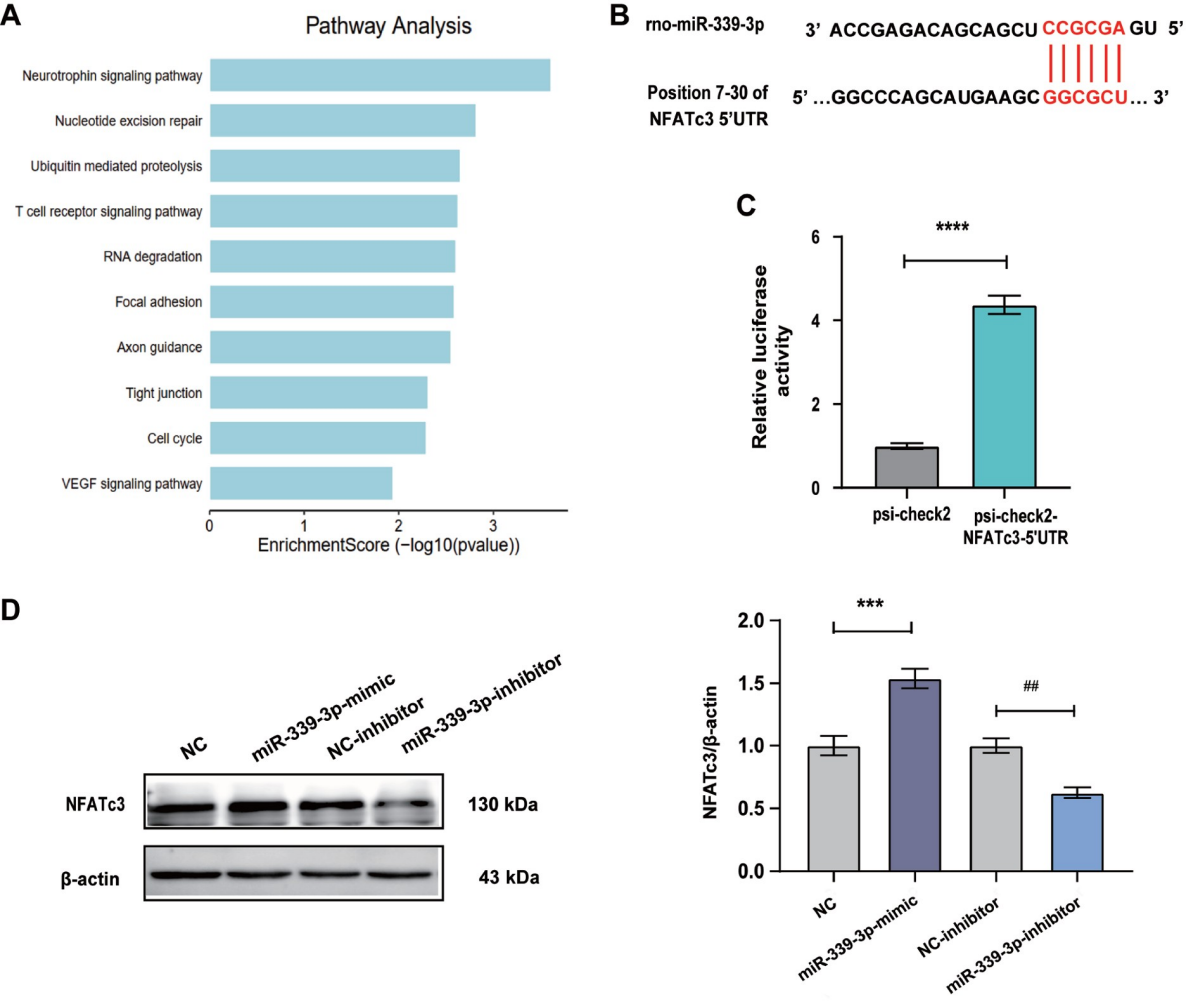
Figure 4 The binding sites of miR-339-3p and the 5′UTR of NFATc3 were predicted by bioinformatics analysis and verified by dual-luciferase reporter assay (A) KEGG analysis of the mRNAs whose 5′UTR could bind to miR-339-3p. (B) Prediction of the binding site between miR-339-3p and the 5′UTR of NFATc3 using the miRwalk website. The dual-luciferase reporter assay was used to verify the binding of miR-339-3p and the 5′UTR of NFATc3. (C) The mimic and inhibitor of miR-339-3p were used to upregulate and downregulate the expression of miR-339-3p in VSMCs, respectively, and the protein expression of NFATc3 was detected by western blot analysis. Data are presented as the mean±standard error. n=3 or 6. ** P<0.01, *** P<0.001, and **** P<0.0001.

How does AT1-AA lead to increased levels of IL-6, IL-1β and TNF-α proteins? It is known that miRNA binding to the 5′UTR of mRNAs can promote the translation of mRNAs. We used the miRwalk database to predict the target genes of miR-339-3p targeting mRNA 5′UTR. KEGG analysis of predicted proteins showed a significant relationship between T cell receptor signaling and inflammation ( Figure 4A), and analysis of genes in this category showed that NFATc3 could significantly promote the secretion of cytokines such as IL-6, IL-1β and TNF-α. The results showed that miR-339-3p has binding sites in the 5′UTR of NFATc3 mRNA ( Figure 4B), and further detection of the dual luciferase reporter gene showed that they could indeed bind each other effectively ( Figure 4C). In addition, after VSMCs were treated with a mimic of miR-339-3p alone, the level of NFATc3 protein in the cells was significantly increased. In contrast, after treatment of VSMCs with the inhibitor of miR-339-3p, NFATc3 protein levels were found to be reduced in VSMCs ( Figure 4D). These data indicated that miR-339-3p plays a role in promoting the protein expression of NFATc3 by binding with its 5′UTR.

### AT1-AA induces a significant increase in NFATc3 protein expression by upregulating miR-339-3p expression in VSMCs

**Figure 5:**
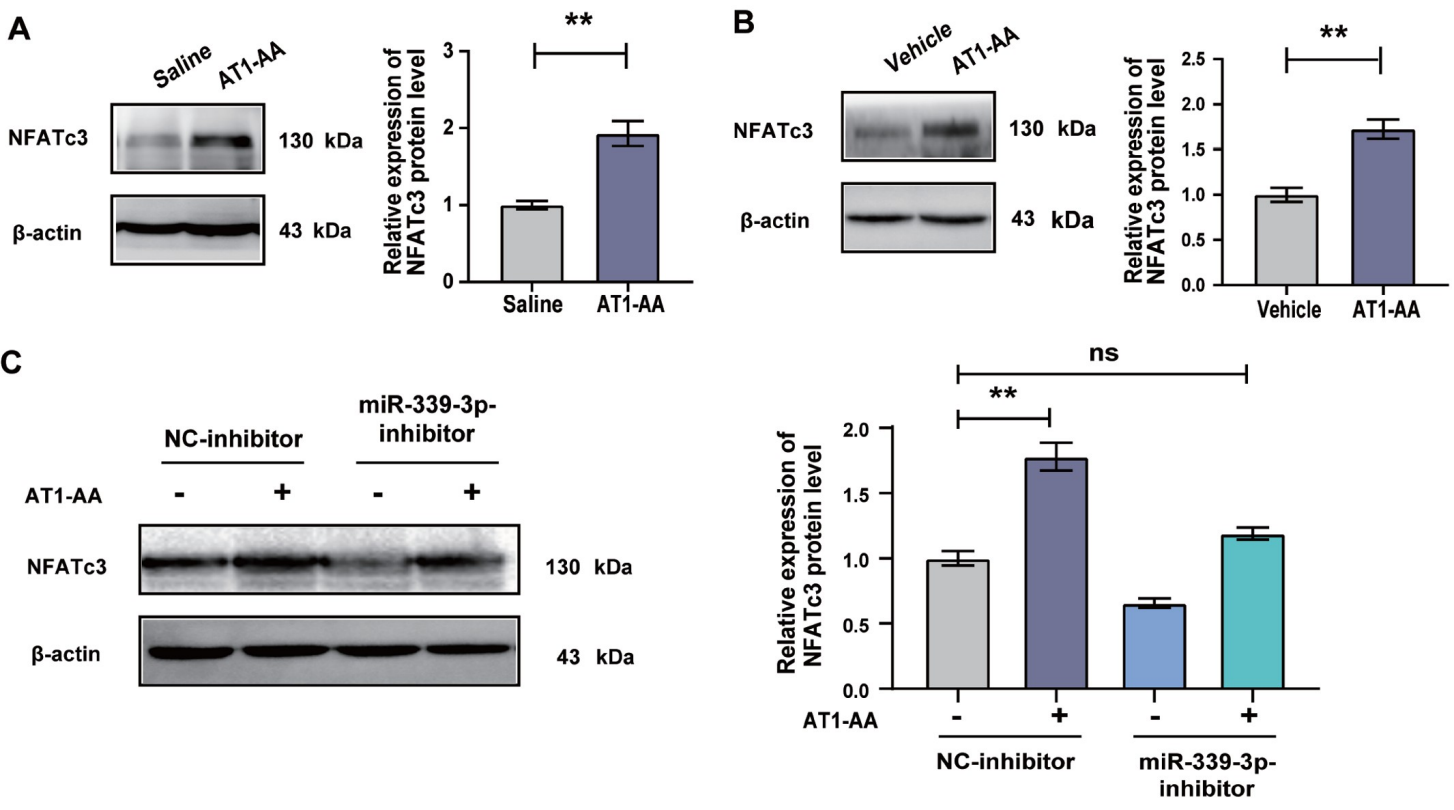
Figure 5 Elevated miR-339-3p is involved in the AT1-AA-induced increase in NFATc3 protein expression The expression of NFATc3 protein in (A) AT1-AA-positive rat thoracic aortas and (B) AT1-AA-treated VSMCs was detected by western blot analysis. (C) VSMCs were treated with the inhibitor of miR-339-3p and then treated with AT1-AA, and the expression of NFATc3 protein was detected by western blot analysis. Data are presented as the mean±standard error. n=5 or 6. ** P<0.01 vs the saline/vehicle/NC-inhibitor group.

Next, we detected the expression of NFATc3 protein and found that the expression of NFATc3 protein was significantly increased in AT1-AA-positive rat thoracic aortas ( Figure 5A) and in VSMCs treated with AT1-AA, compared with the controls ( Figure 5B). VSMCs were treated with the inhibitor of miR-339-3p and then treated with AT1-AA. Finally, it was found that AT1-AA could not promote the increase in NFATc3 protein expression ( Figure 5C). The above results indicated that the increased expression of miR-339-3p was a key factor for AT1-AA to upregulate NFATc3 protein expression.

## Discussion

Vascular inflammation plays a crucial role in the occurrence and development of various cardiovascular diseases. The inflammatory response following RAAS activation underlies a variety of physiological and pathological processes [11]. Stimulation of the angiotensin type II-1 (AT1) receptor triggers nearly all the classic effects of the RAAS, including proinflammatory effects [12]. Studies have reported that AT1-AA can regulate the upregulation of the proinflammatory factors IL-1β and IL-6. This study found that, regardless of the tissue level or the cellular level, the inflammatory factor TNF-α was highly expressed. In addition, IL-6 and IL-1β were also highly expressed in the thoracic aorta vascular tissue of AT1-AA-positive rats and in AT1-AA-treated VSMCs. Dysregulation of persistent synthesis of IL-6 has pathological effects on chronic inflammation and autoimmunity [13]; for example, IL-6 induces overproduction of VEGF, leading to enhanced angiogenesis and increased vascular permeability [14]. IL-1β has strong proinflammatory activity and can induce the release of various proinflammatory mediators, such as cytokines and chemokines [15], exerting multiple effects on various cells and ultimately leading to a wide range of inflammatory events. TNF-α is known for its proinflammatory activity [16], and dysregulation of TNF-α is associated with a variety of pathological conditions, such as infections, autoimmune diseases [17], atherosclerosis [18], and inflammatory bowel disease [19].

miRNAs have emerged as important regulators in inflammation processes [20]. Studies have shown that miR-339-3p is closely related to aneurysm expansion in cardiovascular disease [8] and cell inflammation in acute pancreatitis [9]. We found that the changes in miR-339-3p content in AT1-AA-positive rat thoracic aortas and AT1-AA-treated VSMCs were significantly higher than that in saline group rat thoracic aortas and vehicle group VSMCs. miRNAs, lipids, proteins and other nucleic acids are the main components of exosomes [21], of which miRNAs are the most common [22]. Exosomes are present in the serum/plasma and almost all other body fluids [23], and studies have shown that exosome secretion can affect physiological functions as well as pathophysiological processes, including cardiovascular disease [22]. In this study, we found that when the expression of miRNAs in cells changed, the corresponding miRNAs in exosomes also showed the same trend of change, but the change range was larger [24]. Therefore, as we expected, miR-339-3p expression in serum exosomes of AT1-AA-positive rats established earlier was also significantly higher than that of control rats.

To further explore the possible mechanism by which elevated expression of miR-339-3p induced by AT1-AA promotes vascular inflammation, we used miRwalk database and predicted that miR-339-3p could bind to the 5′UTR of mRNAs and then analysed it by KEGG. The NFAT family contained in the T-cell receptor pathway is closely related to vascular inflammation. The nuclear factor of activated T cells (NFAT) family was originally discovered in T cells and is involved in regulating the immune system, inflammatory response, angiogenesis, heart valve formation, myocardial development and other physiological processes [25]. We screened mRNAs whose 5′UTR can be bound by miR-339-3p and found that the T-cell receptor signaling pathway category includes the NFAT family. NFAT is expressed on the promoters/enhancers of various inflammatory cytokine genes and can regulate the expressions of corresponding inflammatory cytokines [26]. The NFAT family contains five calcium/calcineurin-dependent transcription factors, including NFATc/2/c1, NFATp/1/c2, NFAT3/c4, NFAT4/c3 and NFAT5 [27]. NFATc3, the most important member of the NFAT family [28], is involved in the pathogenesis of various inflammatory pathologies [25] and plays a key role in cytokine secretion [29]. For example, NFATc3 transcriptionally regulates the expression of tumor necrosis factor alpha (TNF-α), promoting the progression of LPS-induced acute lung injury [30]. In addition, NFATc3 can also promote the expressions of IL-6 [31] and IL-1β [26]. In this study, we observed that the expression of NFATc3 protein was significantly increased after treatment with AT1-AA.

It is generally believed that miRNAs can directly mediate posttranscriptional gene silencing (PTGS) in the cytoplasm, and the pathway is only the complementary sequence of the 6 nt seed sequence of miRNAs and the 3′-untranslated region (UTR) of the target mRNA [32]. Studies have also shown that miRNAs can target the 5′UTR of mRNAs to enhance regulation and increase protein levels [33]. Bioinformatics analysis showed that miR-339-3p has a binding site in the 5′UTR of NFATc3 mRNA, and the luciferase reporter assay confirmed that the two can bind effectively. Elevated miR-339-3p can increase NFATc3 protein expression, leading to an enhanced inflammatory response.

In summary, we explored the roles of miR-339-3p and NFATc3 in AT1-AA-stimulated inflammatory changes in vascular tissue and VSMCs. We found that miR-339-3p was upregulated in AT1-AA-positive rat thoracic aortas and in AT1-AA-treated VSMCs, and miR-339-3p promoted the increased expression of the inflammatory cytokines IL-6, IL-1β and TNF-α in AT1-AA-stimulated VSMCs. Bioinformatics analysis showed that the 5′UTR of NFATc3 mRNA is the direct target of miR-339-3p in VSMCs. The inflammatory response of miR-339-3p to AT1-AA-induced VSMCs was achieved by targeting NFATc3 and increasing its protein expression, thereby promoting the secretion of inflammatory cytokines. Taken together, these findings suggest that miR-339-3p targets NFATc3 as a positive regulator of the inflammatory response in VSMCs.
